# Supplementary material for: Molecular insights into heart field-specific cardiomyocyte differentiation - A computational study
Source: PLoS One. 2026 Jan 5;21(1):e0340054. doi: 10.1371/journal.pone.0340054 (PMC12768282; doi:10.1371/journal.pone.0340054)
Supplement: S1 Table — The listed sources form the basis of the interactions that make up Fig 4. (DOCX) [file pone.0340054.s001.docx]

**S1 Table. Extended evidence of the connections constituting the cardiomyocyte subtype GRN.** The listed sources form the basis of the interactions that make up Fig 4.

| **Connection** | **Literature** |
| --- | --- |
| GATA4/6 − > HAND2 | In CMs derived from human induced pluripotent stem cells, GATA6 and GATA4 directly activate HAND2 expression [1]. |
| GATA4/6 − > HEY2 | In mice cells, GATA proteins (together with TBX20 ) enhance HEY2 expression in ventricular CMs [2]. |
| HAND2 − > IRX4 | In mice cells, IRX4 expression is lost by HAND2 (with/or NKX2.5) knockout ventricular CMs [3]. |
| IRX4 − > MYL2 | IRX4 contributes to activating ventricular genes and suppressing atrial genes [4]. |
| IRX4 − > HAND2 | IRX4 activates both HAND1 and HAND2 [5]. IRX4 also activates HAND1 [3]. |
| RA − > NR2F2 | In atrial cells derived from human induced embryonic stem cells, COUP-TFII, a transcription factor encoded by the NR2F2 gene, is robustly upregulated in response to RA during directed atrial differentiation [6]. |
| NR2F2 −\| IRX4 | In mice cardiac cells, COUP-TFII represses IRX4 gene expression in CMs via direct binding to COUP-TFII response elements at the IRX4 genomic loci [5]. |
| NR2F2 −\| MYL2 | In mice cardiac cells, COUP-TFII represses MYL2 gene expression through binding to multiple chromatin sites [5]. |
| NR2F2 −\| HEY2 | In mice cardiac cells, COUP-TFII represses HEY2 gene expression in CMs via direct binding to COUP-TFII response elements at the HEY2 genomic loci [5]. |
| NR2F2 − > MYL7 | In mice cardiac cells, COUP-TFII binds to genomic loci of MYL7, and expression is lost in COUP-TFII knock-out cells [5]. |
| HEY2 −\| MYL7 | In mice cells, ectopic MYL7 (and other atrial genes) expression is observed in HEY2 knockout ventricles [3,5,7,8]. |
| Notch − > HEY2 | In mice cells, expression of HEY2 is increased by NOTCH signalling [9]. |

**References**

1. Sharma A, Wasson LK, Willcox JAL, Morton SU, Gorham JM, DeLaughter DM, et al. GATA6 mutations in hiPSCs inform mechanisms for maldevelopment of the heart, pancreas, and diaphragm. Elife. 2020;9:e53278.

2. Ihara D, Watanabe Y, Seya D, Arai Y, Isomoto Y, Nakano A, et al. Expression of Hey2 transcription factor in the early embryonic ventricles is controlled through a distal enhancer by Tbx20 and Gata transcription factors. Dev Biol. 2020;461(2):124–31.

3. Ng SY, Wong CK, Tsang SY. Differential gene expressions in atrial and ventricular myocytes: insights into the road of applying embryonic stem cell-derived cardiomyocytes for future therapies. American journal of physiology-Cell physiology. 2010;299(6):C1234–C1249.

4. England J, Loughna S. Heavy and light roles: myosin in the morphogenesis of the heart. Cellular and Molecular Life Sciences. 2013;70:1221–39.

5. Wu S pin, Cheng CM, Lanz RB, Wang T, Respress JL, Ather S, et al. Atrial identity is determined by a COUP-TFII regulatory network. Dev Cell. 2013;25(4):417–26.

6. Stefanovic S, Zaffran S. Mechanisms of retinoic acid signaling during cardiogenesis. Mech Dev. 2017;143:9–19.

7. Xin M, Small EM, Van Rooij E, Qi X, Richardson JA, Srivastava D, et al. Essential roles of the bHLH transcription factor Hrt2 in repression of atrial gene expression and maintenance of postnatal cardiac function. Proceedings of the National Academy of Sciences. 2007;104(19):7975–80.

8. Koibuchi N, Chin MT. CHF1/Hey2 plays a pivotal role in left ventricular maturation through suppression of ectopic atrial gene expression. Circ Res. 2007;100(6):850–5.

9. Miao L, Li J, Li J, Tian X, Lu Y, Hu S, et al. Notch signaling regulates Hey2 expression in a spatiotemporal dependent manner during cardiac morphogenesis and trabecular specification. Sci Rep. 2018;8(1):2678.
