# Supplementary material for: Molecular insights into heart field-specific cardiomyocyte differentiation - A computational study
Source: PLoS One. 2026 Jan 5;21(1):e0340054. doi: 10.1371/journal.pone.0340054 (PMC12768282; doi:10.1371/journal.pone.0340054)
Supplement: S2 Table — The Table includes the description of the S1A) Heart Field specification S1B) Cardiomyocyte specification and S1C) Unified differentiation network. (DOCX) [file pone.0340054.s002.docx]

**S2 Table. Boolean network description of the models.** The Table includes the description of the S2A) Heart Field specification S2B) Cardiomyocyte specification and S2C) Unified differentiation network.

**S2A Table: Boolean network description of heart field specification**

xex WNT (t + 1) = xex WNT (t)
xex BMP2 (t + 1) = xex BMP2 (t)
xBMP2 (t + 1) = ¬xWNT (t) ∧ xex BMP2 (t)
xWNT (t + 1) = xex WNT (t)
xDKK1 (t + 1) = xMESP1 (t) ∨ (xWNT (t) ∧ ¬xBMP2 (t))
xFGF8 (t + 1) = ¬xMESP1 (t) ∧ (xFOXC12 (t) ∨ xTBX1 (t))
xFOXC12 (t + 1) = xWNT (t) ∧ ¬xex WNT (t)
xISL1 (t + 1) = xTBX1 (t) ∨ xMESP1 (t) ∨ xFGF8 (t) ∨ (xWNT ∧ ¬xex BMP2)
xMESP1 (t + 1) = xWNT (t) ∧ ¬xex BMP2 (t)
xNKX25 (t + 1) = (xISL1 (t) ∧ xGATAs (t)) ∨ xTBX1 (t) ∨ xTBX5 (t) ∨ (xMESP1 (t) ∧ xDKK1 (t)) ∨ (xBMP2 (t) ∧ xGATAs (t))
xTBX1 (t + 1) = xFOXC12 (t)
xTBX5 (t + 1) = ¬(xTBX1 (t) ∨ xWNT (t)) ∧ (xNKX25 (t) ∨ xTBX5 (t) ∨ xMESP1 (t)) ∧ ¬(xDKK1 (t) ∧ ¬(xMESP1 (t) ∨ xTBX5 (t)))
xGATAs (t + 1) = xNKX25 (t) ∨ xMESP1 (t) ∨ xTBX5 (t)

**S2B Table: Boolean network description of cardiomyocyte specification**

xGATA4/6 (t + 1) = xGATA4/6 (t)
xNotch (t + 1) = xNotch (t)
xRA (t + 1) = xRA (t)
xNR2F2 (t + 1) = xRA (t)
xHAND2 (t + 1) = xIRX4 (t) ∨ xGATA4/6 (t)
xIRX4 (t + 1) = xHAND2 (t) ∧ ¬xNR2F2 (t)
xMYL2 (t + 1) = xIRX4 (t) ∧ ¬xNR2F2 (t)
xHEY2 (t + 1) = xNotch (t) ∧ xGATA4/6 (t) ∧ ¬xNR2F2 (t)
xMYL7 (t + 1) = xNR2F2 (t) ∧ ¬xHEY2 (t)

**S2C Table: Boolean network description of cardiomyocyte specification**

The unified BN consists of the heart field BN and the cardiomyocyte BN. Additional changes are in bold.

xex WNT (t + 1) = xex WNT (t)
xex BMP2 (t + 1) = 1
xBMP2 (t + 1) = ¬xWNT (t) ∧ xex BMP2 (t)
xWNT (t + 1) = xex WNT (t)
xDKK1 (t + 1) = xMESP1 (t) ∨ (xWNT (t) ∧ ¬xex BMP2 (t))
xFGF8 (t + 1) = ¬xMESP1 (t) ∧ (xFOXC12 (t) ∨ xTBX1 (t))
xFOXC12 (t + 1) = xWNT (t) ∧ xex WNT (t)
xISL1 (t + 1) = xTBX1 (t) ∨ xMESP1 (t) ∨ xFGF8 (t) ∨ (xWNT ∧ ¬xex BMP2)
xMESP1 (t + 1) = xWNT (t) ∧ ¬xex BMP2 (t)
xNKX25 (t + 1) = (xISL1 (t) ∧ xGATAs (t)) ∨ xTBX1 (t) ∨ xTBX5 (t) ∨ (xMESP1 (t) ∧ xDKK1 (t)) ∨ (xBMP2 (t) ∧ xGATAs (t))
xTBX1 (t + 1) = xFOXC12 (t)
xTBX5 (t + 1) = ¬(xTBX1 (t) ∨ xWNT (t)) ∧ (xNKX25 (t) ∨ xTBX5 (t) ∨ xMESP1 (t))
**xGATA4/6 (t + 1) = xNKX25 (t) ∨ xMESP1 (t) ∨ xTBX5 (t)**xNotch (t + 1) = xNotch (t)
xRA (t + 1) = xRA (t)
xNR2F2 (t + 1) = xRA (t)
xHAND2 (t + 1) = xIRX4 (t) ∨ xGATA4/6 (t)
xIRX4 (t + 1) = xHAND2 (t) ∧ ¬xNR2F2 (t) ∧ xNKX25 (t)
xMYL2 (t + 1) = xIRX4 (t) ∧ ¬xNR2F2 (t)
xHEY2 (t + 1) = xNotch (t) ∧ xGATA4/6 (t) ∧ ¬xNR2F2 (t)
xMYL7 (t + 1) = xNR2F2 (t) ∧ ¬xHEY2 (t)
