# Supplementary material for: Molecular insights into heart field-specific cardiomyocyte differentiation - A computational study
Source: PLoS One. 2026 Jan 5;21(1):e0340054. doi: 10.1371/journal.pone.0340054 (PMC12768282; doi:10.1371/journal.pone.0340054)
Supplement: Attachment — Submitted filename: Response to Reviewers.docx [file pone.0340054.s004.docx]

# General

**Actions taken**

- We have added page numbers to the manuscript
- We have added line numbers to the manuscript to allow for pinpointing where things have been changed

# Journal requirements

When submitting your revision, we need you to address these additional requirements.

1. Please ensure that your manuscript meets PLOS ONE's style requirements, including those for file naming.

**Actions taken:**

- Changed level 1 heading to Bold type, 18pt font
- Changed level 2 heading to Bold type, 16pt font
- Changed level 2 heading to Bold type, 14pt font
- Put Table titles above the tables and kept the legends below
- Citing style has been changed to brackets
- Made figure captions appear directly after the paragraph they were first cited in
- Funding information is removed from the Acknowledgements
- Added the supporting information titles to the main manuscript

2. Please ensure that you refer to Figure 1 in your text as, if accepted, production will need this reference to link the reader to the figure.

**Action taken:** Page 2 line 33: Referred to Figure 1

3. Please include a copy of Table 6 which you refer to in your text on page 11.

Due to a numbering mistake, Table 6 was incorrectly called Table 5 in the previous version of the manuscript. With the correct numbering, Table 6 is now included.

**Action taken:** Page 11 line 420: Table 6 is now named correctly

4. Please include captions for your Supporting Information files at the end of your manuscript, and update any in-text citations to match accordingly.

**Action taken:**

- Included captions of the supporting information files at the end of the manuscript
- Renamed the supporting information tables to match PLOS ONE guidelines
- Referenced the supporting information tables in the manuscript accordingly

5. If the reviewer comments include a recommendation to cite specific previously published works, please review and evaluate these publications to determine whether they are relevant and should be cited. There is no requirement to cite these works unless the editor has indicated otherwise.

There have been no recommendations on citation of specific previously published works.

# Reviewer #1

The manuscript presents a computational Boolean network (BN) model that integrates early heart field specification with downstream differentiation of cardiomyocytes (CMs) into atrial and ventricular subtypes. This dual-stage modeling approach is innovative and highly relevant to regenerative medicine, as it attempts to bridge the gap between developmental biology and therapeutic applications. The work is ambitious, clearly structured, and supported by extensive literature. However, while the study is promising, several issues limit its impact in its current form. The manuscript requires major revisions to address weaknesses in biological validation, model justification, and clarity of results presentation.

We thank the reviewer for recognizing the merit of our work and providing feedback to improve our work.

1. The exclusion of key regulators (e.g., HAND1, PITX2, ANF, CX40) undermines the model’s ability to reproduce knockout phenotypes. Please justify these exclusions or consider expanding the network to include them.

We thank the reviewer for the insightful comment. We agree that excluding some of the regulators undermines the model's ability to reproduce certain knockout phenotypes. Our goal with this model was to make a minimal network that describes the differentiation of heart field-specific cardiomyocytes, which includes two developmental lineage decisions. For this goal, some exclusions had to be made.

Some nodes are excluded as they do not have influence on the steady states (e.g. ANF, CX40). With the current model complexity, they are coregulated with other factors in the model and would therefore not influence the behaviour of the model and only serve as a readout. Only when expanding the model complexity, these nodes would reveal additional regulatory mechanisms causing the knockout phenotypes to be better represented, but with our central goal being to describe the two developmental lineages, these nodes are excluded.

Other nodes are excluded as they were not deemed interesting for the developmental lineages we are describing. For example, PITX2 is a regulator expressed in the left side of the heart, and it plays an important role in the patterning and organization of the heart. This factor becomes important when evaluating the heart as a tissue, but the purpose of our model is to showcase the single-cell decisions that cause cells to become heart field-specific cardiomyocytes. PITX2 was therefore left out of the model.

Finally, HAND1 is excluded from the model due to both functional redundancy and restrictions to the Boolean formalism. It is not possible to make HAND1 solely be included in the FHF, as there are no feedback mechanisms after the model reaches a certain steady state, and it should therefore express either HAND1 or HAND2. HAND1 and HAND2 have similar mechanisms and can recover their functions to ensure development, but modeling both is only needed when specifically looking for the effect of knocking out either one. With the main purpose of the model being to describe differentiation in a simplified manner, we chose to exclude HAND1.

In the revised version of the paper, we have justified the exclusion of HAND1 and of atrial and ventricular biomarkers in general, because they can influence the model outcomes. Other exclusions will not be mentioned by name in the manuscript to avoid confusion for readers.

**Action taken:**

- Page 6 line 229: Explained exclusions due to similarity of regulation
  - Multiple biomarkers for these cell fates have been identified, which are mostly coregulated and co-expressed (1). We therefore model these clusters of biomarkers with expression of two representative genes; MYL7 for atrial identities and MYL2 for ventricular identities.
- Page 11 line 409: Explained exclusions because of similarity in mechanism
  - HAND1 and HAND2 exhibit functional redundancy during cardiomyocyte differentiation, with HAND1 compensating for HAND2 loss in the FHF to ensure left ventricular development in vivo (2). Including HAND1 would therefore be redundant for modeling the general mechanisms behind the differentiation, but as a result, the model can not distinguish between right- and left ventricle-specific effects.

2. Reliance on MYL7 as the sole atrial marker is insufficient. Incorporating multiple atrial markers would improve biological accuracy.

Incorporating multiple atrial markers may indeed improve the biological accuracy of the model. A point can even be made that in general adding more and more markers would improve the biological accuracy of the model. Additional markers could for example make the knockouts be captured more accurately. The reason for not including more atrial markers was already briefly touched upon in the response to Reviewer #1 comment 1.

With our aim to model a simple representation of the differentiation steps, we reduced the number of nodes that only had one incoming edge and one outgoing edge. In this case, other atrial markers would have the same purpose as adding MYL7 to the model, just to see if the steady state that the cells reach is of atrial fate or not. Other atrial markers will show up parallel to MYL7, and only in cases of specific knockouts, these other atrial markers start serving a purpose (for example, as seen in the TBX5 knockout (page 13 line 454)). We therefore decided not to incorporate multiple atrial (and ventricular) markers.

**Action taken:**

- Page 6 line 229: Explained better why MYL7 is used as a sole atrial marker in this model
  - Multiple biomarkers for these cell fates have been identified, which are mostly coregulated and coexpressed [6]. We therefore model these clusters of biomarkers with expression of two representative genes; MYL7 for atrial identities and MYL2 for and ventricular identities.
- Page 13 line 473: Changed wording on MYL7 being insufficient as a marker for the TBX5 knockout
  - In the context of this knockout, MYL7 can therefore not represent atrial genes as it does for the wild type.

3. The model only partially reproduces published knockout/overexpression studies. Quantitative validation against transcriptomic datasets (e.g., single-cell RNA-seq of differentiating cardiomyocytes) would significantly strengthen the conclusions.

We agree that quantitative validation against a dataset would strengthen the conclusions. The suggested quantitative validation of our Boolean model would require an experiment that uses a protocol with the exact combinations of signalling factors that we are modelling, which measures gene expression/protein activity at the time points we are investigating, specifically looking at the cell types that we are investigating. To our knowledge, there is no single-cell RNA-seq dataset for the cardiomyocyte differentiation steps that solely uses the combination of signaling factors as in our model.

The closest dataset we could find is GSE239891 from Schmidt et al. 2023. Their experiments use signalling combinations specifically for first and second heart field ventricular cardiomyocytes, but not for atrial cardiomyocytes. They also use additional signaling factors (e.g. BMP4, FGF2, Activin A), hampering direct comparisons in expression of the model compared to their dataset.

Performing the required experiment itself is not something that is in the capacity of our lab, nor is it something we can outsource with the time and budget of this research. Hence, we opted for the qualitative validation through published knockout/overexpression studies. In the revised manuscript, we explain the model validation choices better. We also highlight the quantitative validation against a transcriptomic dataset as a future direction.

**Action taken:**

- Page 4 line 152-165: Explain the made choices for model validation better
  - Boolean networks of gene regulatory networks can be validated internally and externally. When a GRN is generated using data, internal validation is performed by determining the quality of the data, and the performance of the inference algorithm [18]. The networks presented in this paper are constructed from published node interactions (see S1 Table and Herrmann et al. [4]), causing included interactions to already be internally validated. The model can also be validated externally by comparing the model topology to a data-generated BN model, or by comparing model predictions with biological data or observations [18]. A data-generated BN can for example be generated using the R package BoolNet [19]. However, this requires a dataset with inputs and readouts that match the model, which does not exist for the network presented in this paper. Additionally, a data-generated BN is not expected to exhibit biologically plausible steady states that capture interactions across two separate time steps, which is necessary for comparison to the presented BN. The best method for externally validating the presented BN is therefore to compare model predictions to external information not used during the modeling process [18]. The model will be qualitatively validated to see if it can predict expression outcomes of published knockout and overexpression experiments, with the methodology exemplified in the section “Knockouts and overexpression for validation”.
- Page 14 line 531: Highlighted the quantitative validation against a transcriptomic dataset as a future direction.
  - Quantitative comparison of the model predictions against a transcriptomic dataset requires an optimized experiment that uses the same set of signaling factors as in the model, applied to the corresponding cell types. Setting up and optimizing such an experiment might be timely and costly but would significantly strengthen conclusions from the model.

4. Tables summarizing perturbations should include a quantitative measure of agreement (e.g., percentage of expected phenotypes reproduced).

Thank you for your feedback on the interpretability of the table. We agree that for our measure of success, “yes”, “semi”, and “no”, are less insightful than giving a percentage for how many signaling inputs the simulated knockout correctly reproduced. We have therefore replaced the reproducing success with a percentage.

**Action taken:**

- Page 12, line 422 (Table 6): Replaced the success descriptions with percentages

| Succes rate | 100% | 75% | 100% | 50% | 75% | 100% | 75% | 100% |
| --- | --- | --- | --- | --- | --- | --- | --- | --- |

- Page 12, row 429 (Table 6 description): Added a quantitative measure of agreement for the amount of reproduced phenotypes
  - The model reproduced 27 of 32 combinations of signaling and perturbations (~84%).

5. Many findings (e.g., RA as a switch, roles of WNT/BMP) are already well established. The novelty lies in integrating early and late differentiation stages, and this distinction should be clearly emphasized.

Thank you for acknowledging the novelty in our paper. The main focus indeed was to integrate early and late differentiation stages, present an understandable network providing insight into how signaling factors govern the differentiation through these stages, and use these networks to make predictions of resulting cell types based on different combinations of factors. If the novelty was not clearly presented before, we will gladly emphasize it more in the revised manuscript.

**Action taken:**

- Page 2 line 58-62: Highlight the lack of coupling early and late differentiation in previous literature
  - The critical roles that these factors individually play in establishing atrial and ventricular identities are well established. There however, is limited understanding of the emergent lineage decision mechanisms that these factors within GRNs give rise to, particularly governing heart field specification and cardiomyocyte subtype differentiation. Especially how early lineage decisions influence the final phenotype of the cell, remains incompletely characterized.
- Page 3 line 92-94: Highlighted that we couple multiple decision timepoints in our model
  - This framework provides a mechanistic basis for the coupling between early and late-stage molecular regulation that determines cardiomyocyte subtype identity and enables prediction of signaling conditions required to generate heart field-specific cardiomyocyte subtypes in vitro.

6. The interpretation of the “null” state as alternative fates is speculative and requires either supporting evidence or a more cautious presentation.

We agree that the interpretation of the null state as an alternative cell fate is speculative. The reason we suggest that the null state that appears in our simulations might indicate an alternative cell fate, is that BMP signaling is activated in the state, while there are no other cardiac markers activated. As BMP signalling is involved in the differentiation of other mesodermal cell fates, and cells of our model start as mesodermal cells, it suggests that this null state may represent another mesodermal cell fate with markers that we did not include in the model, as we are mainly interested in the heart field-specific cardiomyocytes.

Because there are indeed multiple assumptions in the speculation that the null state actually represents a cell fate and is not just a steady state resulting from other modeling simplifications, we present the interpretation more carefully.

**Action taken:**

- Page 9 line 344 (Table 3 description): Be more careful with the interpretation of the null state
  - The remaining “null” steady state results from the inactivation of GATA4/6, a prerequisite for cardiac differentiation (3), therefore causing no downstream cardiomyocyte marker upregulation. Cells remaining in the “null” state are therefore speculated to be mesodermal progenitors or differentiated to another mesodermal lineage not accounted for in the model structure.
- Page 10 line 362: Similarly, we are more careful with statements about what the model suggests
  - The model suggests that there is a state possible where GATA4/6 is not activated, and no cardiac markers get upregulated. These cells with a “null” state identity are therefore speculated to either be non-differentiated precursor mesoderm cells, of cells that may adopt an alternative non-cardiac cell fate not accounted for in the current network structure.

7. Figures 6–7 (network schematics) are dense and difficult to interpret. Simplify or reorganize them, perhaps highlighting only key regulators while relegating secondary interactions to Supplementary Figures.

Although we agree that the network schematics are dense, they serve the purpose of showcasing the gene regulatory mechanisms behind the differentiation steps. Reducing more key factors would make the networks lose their biological interpretability.

Only using a few markers is something that we do for the tables 2,3 and 4 to improve their interpretability, but we think it is important to showcase all markers in the figures 6 and 7 to allow for an accurate description and understanding of the factors and their interactions that give rise to the dynamics of the model. Similar manuscripts, for example Hermann et al (4), also showcase the entire network that is fed in the model, and we as readers have benefitted from that. We think that our target audience can get value out of the full networks, especially as they are already a simplified showcase.

Although we don’t change the figures themselves, we have changed the figure descriptions so that the figures become easier to interpret.

**Action taken:**

- Page 8 line 303 (Fig 6 description): Wording changes to enhance interpretability
  - The network models differentiation of cardiac precursor cells into FHF (with central regulatory node TBX5 shown in blue) and SHF (with central regulatory nodes ISL1 and TBX1 shown in pink) fates guided by BMP and WNT signaling.
- Page 9 line 321 (Fig 7 description): Wording changes to enhance interpretability
  - In this model, expression of TBX5 promotes FHF identity; ISL1 and TBX1 promote SHF identity, MYL7 expression indicates aCM differentiation and MYL2 expression indicates vCM differentiation.

8. The discussion should better frame the model as a hypothesis-generating tool, not yet predictive enough for guiding clinical differentiation protocols.

We agree that the language for the implications of our model may have been too far-fetched, so we have altered it accordingly.

**Action taken:**

- Page 14 line 525: Reduce overselling of the model by giving more realistic applications
  - The dual-stage integration shows how early signals influence the subtype identities, providing a foundation as a hypothesis-generating tool for understanding the emergence of heart field-specific cardiomyocytes. Insights from the model can aid the development of in vitro differentiation protocols aimed at producing specific CM types.

9. Limitations of the Boolean logic approach (e.g., inability to capture simultaneous MYL2 and MYL7 activation in HEY2 knockout) should be more explicitly acknowledged.

Thank you for highlighting this. We indeed dived deeper into the limitations of the Boolean logic approach in our discussion.

**Action taken:**

- Page 13 line 499: Added a paragraph about the limitations of the Boolean Approach with perspective to the system we are modelling.
  - The Boolean logic approach did however have limitations for modelling heart field specific cardiomyocyte differentiation. Two networks that biologically are consecutive were united and steady states were computed asynchronously. Because of this, nodes determining cardiomyocyte identity are already interacting while the heart field decision is still being made. As a result, cardiomyocyte regulators that are heart field specific (e.g. HAND1) cannot be included in the model. Using Boolean logic also caused effects of some knockouts to not be captured correctly. Some knockouts biologically cause ectopic expression of marker genes in other tissues (e. g. MYL7 expression in MYL2-expressing ventricular cardiomyocytes after HEY2 knockout). The Boolean logic does not allow for a steady state to exist where two separate markers are both expressed in the same cell, and these knockouts will therefore not be successfully represented. Although the Boolean modelling approach allows for insights in the system without detailed data on parameters, it lacks implementation of timing and concentrations which are important properties during differentiation.

10. Provide future directions: validation with transcriptomics, incorporation of temporal/spatial aspects, or hybrid Boolean–ODE modeling.

There are multiple possible future directions based on the objectives and research questions to be answered.

If someone uses the model as a basis for an experimental procedure for the generation of heart field specific cardiomyocytes, that person may need to test different concentrations of the inputs (WNT, BMP, RA), as our Boolean approach only captures “on” and “off”.

Someone looking to further validate the model predictions could start by setting up a single-cell transcriptomic experiment to evaluate if the predicted genes that are “on” and “off” per steady state are accurate, and if the predicted cardiomyocyte differentiation outcomes per WNT and RA input match experimental outcomes.

Finally, if someone wants to use the model as a basis for a larger model with more nodes, or put the model in another modelling formalism, that person should look at how the new nodes interact with existing nodes. Depending on the formalism used, new parameters might need to be added like the strength of interactions. It should then also be re-evaluated if the model still exhibits the same behavior. See also our response to Reviewer #1 point 3.

**Action taken:**

- Page 14 line 525: Highlight validation with transcriptomics as a future direction
  - Quantitative comparison of the model predictions against a transcriptomic dataset requires an optimized experiment that uses the same set of signaling factors as in the model, applied to the corresponding cell types. Setting up and optimizing such an experiment might be timely and costly but would significantly strengthen conclusions from the model.
- Page 14 line 539: Mention extension to other model formalisms as a way to expand the model
  - With integration of new datasets and modelling formalisms, the model can be progressively refined to capture more dynamic, spatial and mechanical aspects of heart development, to bring it closer to applications in disease modeling and cell therapy.

11. Ensure consistency in terminology (“heart field” instead of “heat field” in Table 2 caption).

That was indeed a typo, thank you for noticing!

**Action taken:**

- Page 9 line 342: Added r to “second heart field”

12. References: ensure consistent formatting and check for missing details in some citations.

**Action taken:**

- Added missing information (for example, DOI) to some of the references
- The references have been altered to match the PLOS ONE style

13. Improve figure captions: they should interpret results biologically, not just describe content.

We agree that some of the figures were lacking an additional interpretation of the results. The Fig 5 description lacked biological interpretation in general, so we added additional sentences. The figures showcasing the network interactions (Fig 4,6,7) cannot directly be biologically interpreted. The steady states of these figures (which are given in the tables associated with the model), can be biologically interpreted. We will therefore mention the steady state table associated with the models in the figure description, and add additional biological interpretation to the table descriptions when necessary.

**Action taken:**

- Page 6 line 240 (Fig 4): Added reference to the steady state table
  - The steady states of the cardiomyocyte subtype GRN are provided in Table 1.
- Page 7 line 255 (Table 1): Added sentence to interpret ventricular steady state
  - consistent with a ventricular cardiomyocyte identity as observed in vitro (5,6)
- Page 8 line 288 (Fig 5): Added biological interpretation of the RA concentration dependent subtype specification
  - The graph shows that RA orchestrates steady state node activity and therefore cardiomyocyte subtype identity in a signalling intensity-dependent manner. This is consistent with in vivo and in vitro observations where cardiac subtype fates are dependent on RA concentration (7).
- Page 8 line 306 (Fig 6): Added reference to the steady state table
  - The steady states of the heart field specification GRN are provided in Table 2.
- Page 9 line 325 (Fig 7): Added reference to the steady state table
  - The steady states of the unified GRN are provided in Table 3.

Reviewer #2

 This paper addresses an important problem of predicting heart field of the cardiomyocytes when they are differentiated in vitro. This problem is of general interest to both system biologists in terms of cell-fate modeling and experimentalist working with iPSC-derived cardiomyocytes. However, the manuscript has some major issues detailed below:
We thank the reviewer for recognizing the importance of our work and the feedback in improving our manuscript.

(please note that there were no pages, section, or line numbers, so the page with the abstract was assumed to be 1)

1. Right from the introduction, it is not fully clear if the validation of this model has been given proper weight. It would be useful to provide an introduction to how this type of model framework can be validated. (last paragraph page 3)

There are multiple ways to validate a Boolean network, including internal and external (biological) validation. For internal validation, it has to be evaluated if the interactions themselves are plausible. The connections that make up the network are internally validated as these are all based on published literature (as described in Supplementary Table 1 and Hermann et al. (8)). For external (biological) validation, the goal is to see if the network topology can reproduce experimental observations. For this comment, we assume the reviewer is asking for the biological validation of the model predictions.

The external validation can be done quantitatively by comparing model outputs with a matching experimental datasets. Getting such a dataset would require the collaboration with a group that can specifically reproduce the protocol, which was not part of this work and unfortunately we lack the resources. The validation can also be done qualitatively, by looking if individual predictions match reported experimental observations. As there are no data-sets yet that would match our model inputs, we opted for the qualitative validation (see part “Qualitative validation of the unified Boolean Network). For this, we used attractor analysis and perturbation tests (see Table 3 and Table 4).

To make the validation choices easier to understand to readers, we have added a paragraph how this type of model can be validated, and explained our reasons for choosing our validation method. We also refer to our answer to Reviewer #1 point 3 and Reviewer #2 point 3.

**Action taken:**

- Page 4 line 153: Added a paragraph about validation strategies for a Boolean network of GRN, and why we chose specific methods
  - Boolean networks of gene regulatory networks can be validated internally and externally. When a GRN is generated using data, internal validation is performed by determining the quality of the data, and the performance of the inference algorithm [18]. The networks presented in this paper are constructed from published node interactions (see S1 Table and Herrmann et al. [4]), causing included interactions to already be internally validated. The model can also be validated externally by comparing the model topology to a data-generated BN model, or by comparing model predictions with biological data or observations [18]. A data-generated BN can for example be generated using the R package BoolNet [19]. However, this requires a dataset with inputs and readouts that match the model, which does not exist for the network presented in this paper. Additionally, a data-generated BN is not expected to exhibit biologically plausible steady states that capture interactions across two separate time steps, which is necessary for comparison to the presented BN. The best method for externally validating the presented BN is therefore to compare model predictions to external information not used during the modeling process [18]. The model will be qualitatively validated to see if it can predict expression outcomes of published knockout and overexpression experiments, with the methodology exemplified in the section “Knockouts and overexpression for validation”.

2. The idea behind the model dynamics for this paper is rather interesting, but it would be really nice to provide a bit of background here for the more general audience of PLOSOne. As it is, the paper is not very accessible outside the field of synthetic-biology. (page 4 bottom).

Thank you for identifying that the manuscript was not very accessible. We agree that it is important that general readers can follow along with the modelling choices and which options were available. We therefore added more information on other types of modelling formalisms and why we decided Boolean networks were the most suitable for our aim.

**Action taken:**

- Page 3 line 66: Rewrote paragraph for more background information and making it easier to follow for the more general audience
  - Computational modeling offers a means to investigate the emergent behavior of GRNs. A variety of modeling formalisms is available for modeling biological systems, such as Boolean networks, Bayesian networks, Agent-based models and rule-based models to name a few (as reviewed in (13)). The modeling strategy to use is dependent on the system to model and the type of data available and can be classified between mechanistic and empirical approaches. Mechanistic models can simulate the dynamic behavior of regulatory systems but typically depend on detailed kinetic parameters and large-scale quantitative datasets, which are often unavailable in developmental systems. Empirical models infer network structure from experimental data but mainly capture associations between network components without their direct regulatory relationships (14,15). Boolean network (BN) models offer a practical alternative by representing gene regulatory interactions with binary states and logical rules. (14,15). We can use these Boolean networks to make predictions of cardiac differentiation trajectories and to predict the molecular conditions required for generating heart-field-specific cardiomyocytes, without requiring extensive quantitative data.

3. Page 5 - knockout validation paragraph. This could use some more detail as it wasn't clear from this description how the simulation results would be compared back to the experimental data for proper validation.

Thank you for the valuable feedback. The validation procedure is now explained better.

**Action taken:**

- Page 5 line 172: We rewrote the description of the validation procedure to make it clearer
  - Because no dataset directly matches the model’s inputs with information on node activity, the model topology was qualitatively validated. Literature on knockout and overexpression experiments that influence cardiac development was gathered (see Table 5). These perturbations were then replicated in the model, to see if the model predictions still match experimental outcomes.
  - Gene knockouts were simulated by perturbating the corresponding node value to zero (off-state), while constitutive expression of a gene was modeled by fixing the node to one (on-state). For each perturbation, the model was simulated under the same signaling conditions as in the wild-type simulations (WNT, RA; ON or OFF). It is then identified if the model can still reach the attractor states (that corresponds to a cell type) with the perturbation. These results are compared to the published experimental outcomes. A perturbation is considered to be successfully reproduced by the model if the presence or absence of an attractor state matches the (wet lab) experimentally observed presence or absence of a cardiomyocyte cell type.

4. The equation derivation on page 8, seemed out of context and a bit confusing. It is not clear to a general reader how these equations provide insights into the dynamics. Is something known about this dynamics experimentally? Can the authors provide plots to help visualize what they mean? Figure 5 does not have enough context to understand how this prediction is helpful without something similar from experiments or a more comprehensive explanation.

Boolean networks only have the values 0 or 1. Because retinoic acid signaling can vary in strength in vitro, we want to see how well the model can still predict what happens when we make RA have values between 0 and 1. We explain this in the methodology section, but to make it clearer we added more explanation in the results.

**Action taken:**

- Page 7 line 260: We provide some of the context from the methodology again to make it easier to follow why we make the equations
  - The Boolean formalism only allows for binary states. In cells however, signaling strength can vary and is dose-dependent. To investigate if the cardiomyocyte specification model can predict what happens under varying signaling strengths, the Boolean network was extended into a Boolean network Extension (BNE), following the methodology of (16).
- Page 7 line 266: We add an extra sentence to get the goal of the upcoming sections more clear
  - The activity of nodes is then expressed as functions of ρ, to see how varying levels of RA signaling influences cardiomyocyte identity.

5. The table descriptions sometimes get confusing as they refer to column numbers in tables with split columns (page 10).

This is very understandable, and we agree that it becomes ambiguous which column is actually being referred to. As we already refer to the steady states seen in the table in the paragraph, we changed the column numbers to the column descriptions.

**Action taken:**

- Page 10 line 350: Changed the column number to descriptions of the column.
  - In the model, the steady state representing FHF-derived ventricular CMs (column FHF vCM) exhibits TBX5 activity, consistent with left ventricular identity (16,17). Conversely, SHF-derived ventricular CMs (column SHF vCM) exhibit TBX1 expression, corresponding to right ventricular identity (16,17).

6. The abbreviations in the tables should be more consistently defined (example aCM is not defined in the table on page 10).

The abbreviations are provided in the text itself, but indeed not again in the tables. We therefore now included the full name, as well as its abbreviation, in the figure description.

**Action taken:**

- Page 9 line 342: Write the abbreviations in full name
  - The steady states in columns three to six correspond to biologically relevant CM subtypes: atrial cardiomyocytes (aCM) and ventricular cardiomyocytes (vCM) derived from either the first (FHF) or second heart field (SHF).

7. Table 3 has one of the most interesting results of the paper, showing an ability to predict cell populations. It should be possible to compare these results to results from single cell RNA-seq experiments, but there is no mention of validating the model in this way. This would be an actual quantitative validation, which would be very valuable, and should be discussed in length. (page 11)

We fully agree that a comparison to single-cell RNA-seq experiments would strengthen the validation of this model. However, as also mentioned in the comments of Reviewer #1 point 3 and Reviewer #2 point 1, no single cell RNA-seq experiment currently matches the inputs of our model with the time-based readouts needed for the validation. Performing the experiment ourselves is also not something that is in the capacity of our lab, nor is it something we can outsource within the time and budget of this study. We can therefore only highlight why we chose the current method of validation, and mention the quantitative validation as a future direction.

**Action taken:**

- Page 4 line 152: Added paragraph about the selection of the validation method
  - Boolean networks of gene regulatory networks can be validated internally and externally. When a GRN is generated using data, internal validation is performed by determining the quality of the data, and the performance of the inference algorithm [18]. The networks presented in this paper are constructed from published node interactions (see S1 Table and Herrmann et al. [4]), causing included interactions to already be internally validated. The model can also be validated externally by comparing the model topology to a data-generated BN model, or by comparing model predictions with biological data or observations [18]. A data-generated BN can for example be generated using the R package BoolNet [19]. However, this requires a dataset with inputs and readouts that match the model, which does not exist for the network presented in this paper. Additionally, a data-generated BN is not expected to exhibit biologically plausible steady states that capture interactions across two separate time steps, which is necessary for comparison to the presented BN. The best method for externally validating the presented BN is therefore to compare model predictions to external information not used during the modeling process [18]. The model will be qualitatively validated to see if it can predict expression outcomes of published knockout and overexpression experiments, with the methodology exemplified in the section “Knockouts and overexpression for validation”.
- Page 5 line 172: Explain the reason for validating the model qualitatively
  - Because no dataset directly matches the model’s inputs with information on node activity, the model topology was qualitatively validated.
- Page 14 line 531: Highlighted the quantitative validation against a transcriptomic dataset as a future direction.
  - Quantitative comparison of the model predictions against a transcriptomic dataset requires an optimized experiment that uses the same set of signaling factors as in the model, applied to the corresponding cell types. Setting up and optimizing such an experiment might be timely and costly but would significantly strengthen conclusions from the model.

8. The validation on page 12 is purely qualitative, and the logic for why it is so instead of a quantitative validation is confusing. It's confusing that "light green indicates that a cell type is predicted to appear" for experimental work. Is it dominant? What does it mean predicted in such a context?

We apologize for the confusion, our description of the validation may have been insufficient (also see Reviewer #2 comment 3). We improved the description of the validation in the methodology section and altered it in the Table 6 description for easier interpretation.

**Action taken:**

- Page 5 line 170: Improved explanation of the methodology for the validation
  - Because no dataset directly matches the model’s inputs with information on node activity, the model topology was qualitatively validated. Literature on knockout and overexpression experiments that influence cardiac development was gathered. These perturbations were then replicated in the model to see if the model predictions still match experimental outcomes.
  - Gene knockouts were simulated by perturbing the corresponding node value to zero (off-state), while constitutive expression of a gene was modeled by fixing the node to one (on-state). For each perturbation, the model was simulated under the same signaling conditions as in the wild-type simulations (WNT, RA; ON or OFF). It is then identified if the model can still reach the attractor states (which corresponds to a cell type) with the perturbation. These results are compared to the published experimental outcomes. A perturbation is considered to be successfully reproduced by the model if the presence or absence of an attractor state matches the (wet lab) experimentally observed presence or absence of a cardiomyocyte cell type.
- Page 12 line 420 (Table 6 description): The Table description has been altered so it is easier to understand what the table means
  - Cardiomyocyte subtypes appear under specific signaling combinations (column 1). Literature reports that under genetic perturbations, some cardiomyocyte subtypes do not appear anymore (as seen in Table 5). The table compares expected differentiation outcomes from literature (Exp) with simulated steady states (Sim) for seven genetic perturbations. Light green in the expected column (Exp) indicates that a cell type is reported to appear in the literature with that signaling combination (WNT, RA), while white denotes its absence. In the simulated column, dark green indicates that a steady state for a cell type is reached during simulations with that signaling combination (WNT, RA), whereas gray indicates that the steady state is not reached. If both the expected and simulated column agree that a cell type does or does not appear under the signalling combination, the simulation is considered a success. Mismatches between expected and simulated outcomes are unsuccessful and marked with a cross. The model reproduced 27 of 32 combinations of signaling and perturbations (~84%).

9. Page 13 - it would be nice if the discussion section presented this work in the context of other works that predicted cell fate during differentiation.

We understand that general readers may not have an overview Boolean models in the context of predicting cell fate, and what alternatives there are. We therefore highlight some publications where Boolean models have previously been used for modelling cell fate, and in what ways our model has similarities and differences. There are also modelling formalisms besides Boolean to describe and predict cell fate in response to signalling cues, so we mention examples of models in other formalisms. We additionally compare our model with the models from these other formalisms. By placing our model in context of other Boolean models, and in the context of similar models in other formalisms, we give the reader a better idea how our model and modelling approach compares with the state of the art.

**Action taken:**

- Page 14 line 511: Added Alinea comparing the presented model with other Boolean models and models in other formalisms.
  - Boolean networks have previously been used for understanding the regulatory mechanisms behind differentiation of multiple different cell types. This is done either by inferring GRN structure of a system from a single cell or bulk dataset (RNA-seq or qRT-PCR) with an algorithm (e.g. [39–41]), or by manually assigning Boolean rules to literature-reported observations (e.g. [4,15,42,43]). The approach in this manuscript is similar to the second category, but extends it in two ways. Instead of modelling a single lineage decision, we couple two consecutive differentiation stages in one Boolean Network, to see how heart field-specific cardiomyocytes can be generated. We also couple our GRN to external signaling inputs to gain additional insights on how the GRN activities can be influenced to steer differentiation towards a specific cell type. Other modelling modalities can also be used to predict signaling combinations with additional information on the effects of signaling strength (such as timed automata, e.g. [44]; and ODE-based modelling, e.g. [45,46]), something Boolean network predictions lack. Still, these methods require a tremendous amount of data for parameter optimization, whereas here it's shown that Boolean Networks can already provide applicable insights from a simple dynamic approach.

10. The first paragraph of model validation on page 13 is overstated - only 3 of the 7 fully agreed (and qualitatively at that).

We agree with this feedback, the statements on the validation were overstated. We have presented the statements more carefully.

**Action taken:**

- Page 13 line 463: Make the text overstate validation results less.
  - Three out of seven perturbations were fully reproduced, such as the suppression of atrial fate upon COUP-TFII knockout (26) and repression of atrial gene expression under HEY2 overexpression (1,27,28). Two perturbations were partly reproduced, and two perturbations were not captured. These results support the model’s capacity to capture core regulatory dependencies driving CM subtype identity.
  - The discrepancies in some of the model predictions reflect either missing regulatory links or simplifications inherent in a Boolean logic framework.

Minor
- There are two Table 2s and a missing Table 6. It was rather hard to know what referenced what as a result

We apologize for the wrong numbers of the tables. We have fixed the numbering of the Tables, so that there now if only one Table 2 and Table 6 is corrected again.

**Action taken:**

- Page 9 line 327; 337, Page 10 line 346: Table 2 has been corrected to Table 3
- Page 10 line 371, 372, 374, 344, 378, 381: Table 3 has been corrected to Table 4
- Page 11 line 413, Page 12 line 421: Table 4 has been corrected to Table 5
- Page 11 line 418: Table 5 has been corrected to Table 6

- Page 10 last line, the periods were probably meant to be commas in the large numbers of simulations.

We indeed missed this mistake. The periods were all meant as commas to denote groups of three digits, so we will correct this accordingly.

**Action taken:**

- Page 10 line 369, 373, Table 4: Periods have been changed to commas
